# When Figurative Language Goes off the Rails and under the Bus: Fluid Intelligence, Openness to Experience, and the Production of Poor Metaphors

**DOI:** 10.3390/jintelligence9010002

**Published:** 2021-01-05

**Authors:** Paul J. Silvia, Roger E. Beaty

**Affiliations:** 1 Department of Psychology, University of North Carolina at Greensboro, Greensboro, NC 27402, USA; 2 Department of Psychology, Pennsylvania State University, 140 Moore Building, University Park, PA 16801, USA; rebeaty@psu.edu

**Keywords:** metaphor, creativity, figurative language, fluid intelligence, openness to experience

## Abstract

The present research examined the varieties of poor metaphors to gain insight into the cognitive processes involved in generating creative ones. Drawing upon data from two published studies as well as a new sample, adults’ open-ended responses to different metaphor prompts were categorized. Poor metaphors fell into two broad clusters. Non-metaphors—responses that failed to meet the basic task requirements—consisted of “adjective slips” (describing the topic adjectivally instead of figuratively), “wayward attributes” (attributing the wrong property to the topic), and “off-topic idioms” (describing the wrong topic). Bad metaphors—real metaphors that were unanimously judged as uncreative—consisted of “exemplary exemplars” (vehicles that lacked semantic distance and thus seemed trite) and “retrieved clichés” (pulling a dead metaphor from memory). Overall, people higher in fluid intelligence (Gf) were more likely to generate a real metaphor, and their metaphor was less likely to be a bad one. People higher in Openness to Experience, in contrast, were more likely to generate real metaphors but not more or less likely to generate bad ones. Scraping the bottom of the response barrel suggests that creative metaphor production is a particularly complex form of creative thought.

## 1. Introduction

Like their students, college professors dread some classes. In Mathematics departments, according to our shadowy network of informants, that class is “Business Calculus.” If you are wondering how bad teaching a low-level math class to business students could be, one of our math colleagues once quipped, “Business Calculus is the battlefield medicine of teaching: you’re mostly trying to prevent fatalities.” That quote conveys a lot—not only about the meager enthusiasm that marketing students have for derivatives, but also about the creative and expressive power of metaphor. A clever metaphor like that carries a lot of meaning: it expresses a sense of the class in a way that mere adjectives—describing the class as *terrible*, *awful*, *grim*, *harrowing*, or *traumatic*—simply cannot.

How do people come up with metaphors like that? Psychological research has largely focused on how people read, interpret, and understand metaphors, but a growing body of work has explored how people generate metaphors, ranging from conventional, idiomatic metaphors (“my job is a jail”; Pierce and Chiappe 2009) to clever, creative metaphors. To date, research has primarily established that the process of generating creative metaphors involves executive abilities, such as fluid intelligence, as well as personality traits that foster creative thinking, such as Openness to Experience and need for cognition (Beaty and Silvia 2013; de Barros et al. 2010; Primi 2014; Skalicky 2020).

In the present research, we sought to illuminate the mechanics of metaphor production by focusing on the bad ones. Failed attempts at creativity can offer unique insights into the cognitive processes that underlie clever and original ideas. Using two prior datasets (Beaty and Silvia 2013; Silvia and Beaty 2012) as well as a new sample, we extracted the worst metaphors and examined them to understand the ways that metaphors go off the rails. As we will see, an attempt to come up with a creative metaphor can go awry in many ways, and these misfires reveal much about metaphor creation. Two key predictors of metaphor creativity—fluid intelligence and Openness to Experience—were included as predictors to sharpen our understanding of the different kinds of uncreative responses.

### 1.1. Crafting Creative Metaphors

Little is known about how people create metaphors, but theories of metaphor comprehension can be repurposed for insights into how people generate metaphors (Silvia and Beaty 2012). In Glucksberg’s (2001, 2003, 2008) *property attribution model*, metaphors involve asserting that a *topic* has an attribute of a *vehicle*. The comedian Martin (2006), for example, once quipped, “Glitter doesn’t go away. Glitter is the herpes of crafts supplies.” In this example, Martin describes glitter (the topic) by asserting that it has an attribute of herpes (the vehicle). People understand the metaphor by creating a superordinate category, known as an *attributive category*. This category must contain both the topic and vehicle, but they can be in different regions. For an apt metaphor, the vehicle should be an exemplar of the category (“aversive things you can never get rid of”), but the topic need only be a plausible member of it.

This example illustrates some key points about both the comprehension and production of metaphors. First, for creative metaphors, the semantic distance between the topic and vehicle can be vast. Unlike conventional metaphors and idioms, creative metaphors are unfamiliar or wholly novel, so they cannot be produced or understood through simple recognition or retrieval. Second, much of the action for creative metaphors is in the attributive category. For a metaphor to be apt and interesting, someone must identify a suitable category that contains exemplary vehicles and could plausibly include the topic.

And third, generating a clever metaphor looks hard—the cognitive processes are abstract and resource-intensive. To create a good metaphor, people must first select a topic (glitter) and then identify the feature they want to attribute or emphasize, which may involve creating an ad hoc category (e.g., “things that are irritating and impossible to get rid of”). This mental category must be strategically searched for exemplars, which are sorted and rejected (e.g., mosquitoes and caterwauling alley cats do not seem to work as well). Throughout, people must maintain the attributive category and not be distracted by irrelevant vehicle features, including highly salient features that are immaterial to the metaphor (e.g., herpes is a viral infection with two simplex variants). And at the end, like all creative ideas, the metaphor must be reworked and crafted so that it “clicks” pragmatically for the discourse context. We are not suggesting that this is a definitive model of steps—creative ideation is rarely so linear—but our general point is that metaphor creation will involve these processes, which collectively imply that metaphor creation is executively demanding.

Thus far, the small literature on creative metaphor production shows a prominent role for intellectual abilities. Some projects measure creative production with a stem completion format, in which people read incomplete sentences and stems, such as “His skin was as brown as _____” (Taylor 1947) or “The camel is the _____ of the desert” (de Barros et al. 2010; Primi 2014), and complete them with one or several apt responses. Other projects create lengthy situational prompts that provide the topic and feature, such as asking college students to describe the most boring class they ever took using a metaphor (Beaty and Silvia 2013; Silvia and Beaty 2012; Skalicky 2020). In both methods, the results are typically scored for overall creative quality, novelty, aptness, or quantity (for methods that request multiple responses).

Regardless of the specific scoring approach, many intelligence factors predict the creative quality of metaphor production. Medium and large effect sizes have been found for fluid intelligence (Gf; Beaty and Silvia 2013; Menashe et al. 2020; Primi 2014; Silvia and Beaty 2012). Effects of crystallized intelligence (Gc) tend to be smaller (Beaty and Silvia 2013), which is surprising for a language-based task but consistent with the executively demanding nature of metaphor creation. The most intriguing effect might be for broad retrieval ability (Gr), which is usually assessed with fluency tasks that assess how well people selectively retrieve information from mental categories (e.g., animals, things that are loud, or words starting with *F*). Gr has had large effects in past work (Beaty and Silvia 2013), consistent with the central involvement of attributive category processes in metaphor. The small neuroimaging literature supports a view of metaphor generation as executively demanding. Frontal and parietal brain regions associated with executive control show increased activation when people produce metaphors in the scanner (Beaty et al. 2017; Benedek et al. 2014b).

Outside of cognitive abilities, the most salient predictor of metaphor creativity is Openness to Experience, one of the major traits of personality (Sutin 2017). Two studies have found medium and large effects of Openness to Experience on metaphor creativity (Beaty and Silvia 2013; Silvia and Beaty 2012). People high in Openness to Experience see themselves as creative people (Karwowski and Lebuda 2016), have artistic interests and creative hobbies (Conner and Silvia 2015), and perform much better on a wide range of creativity tasks, such as divergent thinking (Silvia et al. 2009) and humor production (Nusbaum et al. 2017; Sutu et al. in press). Openness to Experience is worth examining alongside intelligence because it correlates with many cognitive and language abilities and captures motivational aspects of thinking and reasoning (Ackerman and Heggestad 1997; Silvia 2006).

### 1.2. Diving into the Shallow End: The Present Research

To date, the small literature on metaphor creativity shows prominent effects of cognitive abilities and Openness to Experience. So far, however, the focus has been on global outcomes, such as the quantity or holistic quality of a response. The process and cognitive components of metaphor creation have received relatively little attention. A fertile strategy for understanding what goes into making a creative metaphor is to study the ones that went awry. Errors, slips, and mistakes can reveal the essential ingredients of complex cognitive processes, and they are especially useful for studying creative thought. Highly clever, creative ideas tend to be singular, surprising, and novel; bad ideas, on the other hand, are prevalent and alike. They thus are a good place to start for unpacking the main ingredients of creative metaphor production.

In the present research, we recoded and categorized the metaphors from two prior datasets along with one new dataset. The metaphors were first grouped into two broad categories. In our experience with metaphor assessment, particularly poor responses are either *non-metaphors* or *bad metaphors*. Non-metaphors are responses that do not meet the loose task criteria for a credible metaphor. It is rare that participants are stumped and generate nothing, although it does happen. Instead, people generate a response that is not actually a suitable response to the metaphor prompt. What do they come up with instead, and what can these failed attempts illustrate about the process? We classified the non-metaphors to explore subtypes. One particularly interesting kind of non-metaphor is an *adjective slip*. These responses convey the intended meaning but are not figurative. When describing the grossest thing they ever ate, for example, they might say “It was super nasty!” For these responses, instead of identifying an attributive category (“things that taste gross”) and finding a vehicle that exemplifies it, people simply apply the property to the topic adjectivally (“It tasted gross!”). This seems like an instance of the broader family of executive control failures, such as when a task goal slips from one’s mind mid-task (Welhaf et al. 2020).

Unlike non-metaphors, bad metaphors meet the criteria for a figurative metaphor but lack creativity. We classified bad metaphors—the responses that unanimously received the lowest possible creativity score from a set of independent raters—to see what kinds emerged and how prevalent they were. Based on our past experience administering and scoring metaphor tasks, we expected two subtypes to be prominent. Some bad metaphors lack semantic distance. Consider a “gross food” response like, “Drinking a kale smoothie was like drinking vomit.” The topic (kale smoothies) and vehicle (vomit) are both members of the attributive category (“drinks that taste gross”). The response has all the features of a metaphor: the topic is a plausible category member, and the vehicle is an exemplary member. But the response feels trite and obvious because the vehicle is too exemplary. If a large sample of people were given an admittedly odd verbal fluency task that asked them to list gross, nasty liquids, vomit would probably be among the most frequent early responses. For such metaphors, the lack of semantic distance makes the response apt and easy to understand, but people do not experience it as fresh, interesting, or clever.

Other bad metaphors are canned idioms. In other domains of creativity assessment, bad responses are often “old ideas” that are directly retrieved from memory (Benedek et al. 2014a; Gilhooly et al. 2007). The property people are given to attribute, such as *boring*, can activate salient, idiomatic phrases (e.g., “like watching paint dry”). Many people will stop the creative ideation process once they reach the first seemingly suitable response, and the metaphors that come to mind first are often canned, conventional expressions. In short, we expect that two kinds of bad metaphors will stand out: semantically close vehicles, and salient idioms retrieved directly from memory.

In addition to classifying the metaphors, we explored the roles of fluid intelligence (Gf) and Openness to Experiences. Past work has examined them as predictors of metaphor creativity across the full spectrum of low to high creativity. In these analyses, we focused on how well they predicted the probability of generating non-metaphors and bad metaphors. Although these traits surely aid creativity by fostering good responses, much of their benefit might come from helping people avoid the most obviously bad ones.

## 2. Materials and Methods

### 2.1. Participants

The participants had taken part in one of three studies of creative metaphor production. Two datasets come from published research that has been recoded and reanalyzed. The first dataset (Silvia and Beaty 2012) provided responses for the “boring class” prompt, and it had a final sample of 123 (86 women, 37 men) after omitting 10 participants (9 non-native speakers, 1 software failure). The second dataset (Beaty and Silvia 2013) provided responses for the “gross food” prompt, and it had a final sample of 201 (141 women, 60 men) after omitting 21 participants (9 non-native speakers, 12 inattentive and disengaged). See the prior articles for additional demographic details.

The third dataset provided responses for the “terrible movie” prompt and has not been reported previously. It had a final sample of 137 undergraduate students—102 women, 35 men—from the University of North Carolina at Greensboro (UNCG; age *M* = 19.42, *SD* = 3.55, range from 18 to 51). Students volunteered to participate and received credit toward a research option in a psychology class. Based on self-reports, the sample’s racial and ethnic composition was diverse—African American (35%), Asian American (10%), European-American (50%), and Hispanic/Latinx (10%)—and people could select several categories or decline to select any. Several people had been excluded from a larger sample. As in our past work, non-native speakers of English were omitted (*n* = 12); bilingual speakers of Spanish and English were included in the final sample. Three additional people were excluded for not completing the task or based on markers of inattentiveness and disengagement.

### 2.2. Procedure

In all three samples, people took part in groups of 1 to 8. The tasks and surveys were administered using MediaLab v2010. All subjects gave their informed consent for inclusion before they participated in the study. The study was approved and monitored by the UNCG Institutional Review Board.

#### 2.2.1. Metaphor Assessment

Viewed globally, our metaphor task asked people to generate a single creative metaphor, allowed them as much time as they wished, and then coded and rated their response. The task began by defining what metaphors are and giving examples of different kinds, which were described as simple metaphors (e.g., “All the world’s a stage”), similes (e.g., “Justice is like a train that is nearly always late”), and compound metaphors (e.g., “Life is like a box of chocolates: you never know what you’re going to get”). The full instructions are in Appendix A.

Participants expected to create their own metaphors in response to prompts. The software instructions and experimenter emphasized that the goal was to come up with *creative metaphors*, which were described as “something clever, humorous, original, compelling, or interesting.” Just as in other forms of creativity assessment, “be creative” instructions ensure that the researchers and participants have the same understanding of the task (Nusbaum et al. 2014).

The three samples had essentially similar procedures but different sets of metaphor prompts. For breadth and variety, we selected a different metaphor prompt from each sample, referred to as *terrible movie*, *boring class*, and *gross food*. The participants were told:

- Think about the worst movie or TV show you have ever seen. What was it like to watch it? Please describe the experience with a metaphor.
- Think of the most boring high-school or college class that you have ever had: What was it like to sit through? Please describe the experience with a metaphor.
- Think about the most disgusting thing you ever ate or drank. What was it like to eat or drink it? Please describe the experience with a metaphor.

To help get people off the ground, the instructions offered some possible stems (e.g., *If you need a start, you can try a beginning like “That class was…”, “Being in that class was like…”, or “Sitting through that class was like…”*).

#### 2.2.2. Rating the Responses

The creative quality of the metaphors was rated using subjective scoring methods. The raters used a 5-point scale (1 = *not at all creative*, 5 = *very creative*). The terrible movie and boring class prompts had 3 raters, and the gross food prompt had 4 raters. Two raters were identical across all 3 datasets. The raters gave their scores independently of each other and unaware of all other information about the participants. The internal consistency of the ratings was acceptable for the terrible movie (α = 0.74), boring class (α = 0.64), and gross food (α = 0.83) items.

#### 2.2.3. Coding Non-Metaphors

In all samples, we classified each response as being a metaphor or not. Because our prompts were relatively open-ended and encouraged different kinds of metaphor structures, we coded responses as a metaphor (coded 1) if they involved a classic metaphor comparison (*x* is *y*), a simile structure of linking or resemblance (*x* is like *y*), a figurative expression with a topic-figure-ground structure (*x* is like *y*: ground of *z*; Goatly 2011), or an analogical mapping involving figurative speech (*x* is to *y* as *p* is to *q*). In addition, the response was coded as a non-metaphor if it failed to use the proper topic (e.g., a metaphor about gross food had to actually be about food) or if it failed to express the intended meaning (e.g., the gross food had to be described as unpleasant, if only loosely). All other responses were non-metaphors (coded 0).

#### 2.2.4. Coding Real-But-Bad Metaphors

In the next step, all responses that were classified as real metaphors were further classified. To find the worst responses, we identified the responses that were unanimously judged as bad by the raters. Because each rater gave an independent score on a 1–5 scale, we can define the subset of responses that received a “1” from all raters as the “bad metaphors.” The scores for which the raters unanimously gave the lowest possible score were classified as “bad metaphors” (coded as 1); the rest were classified as “not bad” metaphors (coded 0). In short, the responses at the floor made up the bad metaphors, and all other responses, from the barely better to the best ones, were the not-bad ones.

#### 2.2.5. Coding Subcategories

For non-metaphors and bad-metaphors, two people grouped the responses into clusters. The first pass established a preliminary set of subcategories; for the next pass, responses were sorted into the subcategories, and disagreements were resolved via discussion. Concepts from the property attribution model were used as guidance for developing the categories and classifying the responses.

#### 2.2.6. Fluid Intelligence

The three studies assessed fluid intelligence (Gf) using similar and overlapping sets of tasks, all of which have been widely used in our past research (Christensen et al. 2018; Frith et al. in press). Table 1 describes the tasks used in each sample. Reliability, based on coefficient omega, was good in all three samples: terrible movie (ω = 0.67), boring class (ω = 0.75), and gross food (ω = 0.66). For each sample, Gf was specified as a latent variable with the tasks as indicators. We should note that because some samples used more Gf tasks then others, the complexion of the underlying latent variable could vary slightly across the samples.

#### 2.2.7. Openness to Experience

Openness to Experience was assessed using the 12-item scale in the NEO Five Factor Inventory (NEO-FFI; Costa and McCrae 1992), which gives broad coverage of the trait relative to its length (Christensen et al. 2019). People responded to the items using a 5-point response scale (1 = *strongly disagree*, 5 = *strongly agree*), and the items were averaged for an overall score. Internal consistency was good for all three samples: terrible movie (α = 0.78), boring class (α = 0.77), and gross food (α = 0.65).

## 3. Results

### 3.1. Analysis Plan

We conducted three separate sets of analyses, one per sample. For simplicity, they are presented together instead of as separate studies. For each sample, we conducted two univariate models. Because our key outcomes were binary—whether people generated a metaphor or not, and whether the real metaphors were bad or not—they were modeled as categorical using a logistic link. Note that the outcome variables cannot be included in the same model because the responses classified as non-metaphors will not have a bad-metaphor score.

For each sample, Gf was modeled as a latent variable, with its variance fixed to 1, using the Gf tasks as indicators; Openness to Experience was an observed variable. For the two samples with more than three Gf indicators, a CFA found good model fit (Boring Class: χ^2^(9) = 13.74, *p* = 0.132, CFI = 0.958, RMSEA = 0.065 [90% CI: 0.000, 0.131], SRMR = 0.046; Terrible Movie: χ^2^(2) = 4.63, *p* = 0.099, CFI = 0.966, RMSEA = 0.098 [90% CI: 0.000, 0.218], SRMR = 0.030). Gf and Openness to Experience were included as predictors together in the same model. All indicators and predictors were standardized to center them at zero. The raw values had similar distributions across the three samples, consistent with each study drawing from the same student population. The reported regression coefficients are fully standardized. The analyses were conducted in Mplus 8.1 using maximum likelihood with robust standard errors.

### 3.2. Non-Metaphors

We start with the non-metaphors. Across the three samples, a notable proportion of responses were coded as not being a metaphor: 6% (Gross Food), 11.7% (Terrible Movie), and 15.4% (Boring Class). We coded this variable as 0 (non-metaphor) and 1 (real metaphor) and examined how Gf and Openness to Experience predicted the likelihood that people generated a real metaphor.

**Figure 1:**
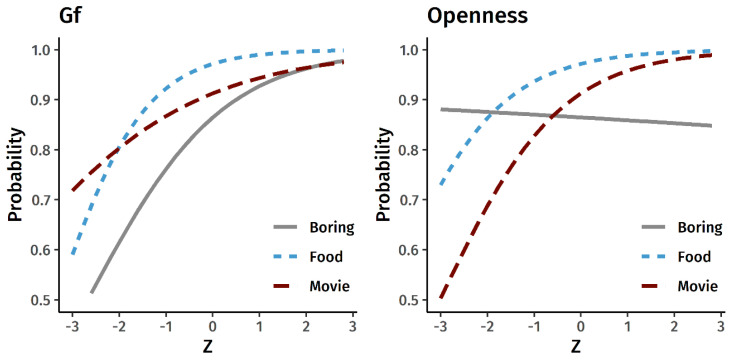
Gf, Openness to Experience, and the probability of generating a real metaphor vs. a non-metaphor. The x-axis represents latent Gf scores and observed Openness to Experience item-average scores in the standardized z-metric.

Figure 1 displays the model-estimated probability of generating a real metaphor as a function of levels of Gf and Openness to Experience. It is apparent that in most cases the slopes are positive: as Gf and as Openness increase, people are more likely to generate a real metaphor instead of a non-metaphor. The effects of Gf were significant for the Boring Class (β = 0.36 [0.08, 0.64], *p* = 0.012) and Gross Food (β = 0.47 [0.19, 0.75], *p* = 0.001) metaphors but not for the Terrible Movie (β = 0.23 [−0.13, 0.60], *p* = 0.212). Likewise, the effects of Openness to Experience were significant for the Gross Food (β = 0.38 [0.14, 0.61], *p* = 0.002) and Terrible Movie (β = 0.38 [0.11, 0.66], *p* = 0.006) metaphors but not the Boring Class (β = −0.03 [−0.24, 0.19], *p* = 0.822).

After classifying the non-metaphors into subtypes, we arrived at an interesting group of subtypes. Table 2 shows the percentages of responses in each subtype; Table 3 provides examples from the data.

Along with the inevitable “Other” category, we found that the non-metaphors could be sorted into a few categories:

- *Adjective Slip.* As expected, many non-metaphors were adjective phrases, not figurative expressions. In these responses, people did not compare the topic to something else or subsume the topic and vehicle in the same category. Although the intended meaning was correct—the gross food was indeed described as gross—people expressed the meaning adjectivally using the defining feature of the attributive category.
- *Wayward Attribute.* Many responses misplaced the vehicle—the response used an incorrect attributive category. For example, such responses did not attribute a proper feature to the topic: a boring class was described as fun or a gross drink was described as fine.
- *Off-Topic Idiom.* Many responses were expressions that had a metaphor structure but were literally off-topic: the metaphor was about something other than the topic in the prompt. These responses were usually idioms, song lyrics, motivational sayings, and other accessible phrases drawn from memory.

In short, the qualities of non-metaphors align well with the concepts in the property attribution model. In some cases, there was a failure of abstract comparison and categorization—people expressed the sense of the topic adjectivally instead of figuratively. In other cases, people failed to use the proper feature (the wrong property was attributed to the right topic) or failed to describe the proper topic.

### 3.3. Bad Metaphors

We next explored the bad metaphors—the responses that were classified as real metaphors but were unanimously given the worst possible score by the independent raters. These bad metaphors were common—13.2% (Terrible Movie), 16.3% (Boring Class), and 58.2% (Gross Food)—especially for the Gross Food prompt. We coded this “bad metaphor” variable as 0 (not bad) and 1 (bad) and examined how Gf and Openness to Experience predicted the likelihood that people generated a bad metaphor.

**Figure 2:**
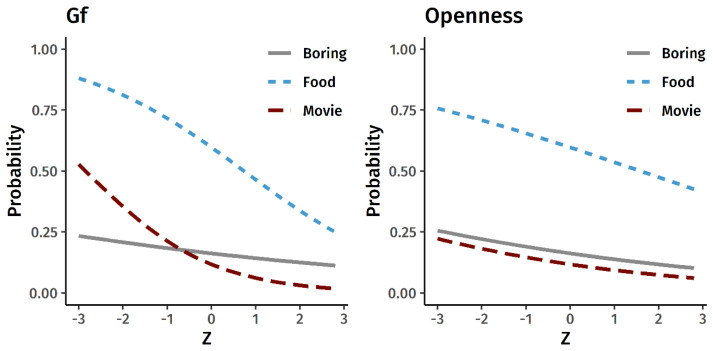
Gf, Openness to Experience, and the probability of generating a bad metaphor. The x-axis represents latent Gf scores and observed Openness to Experience item-average scores in the standardized z-metric.

Figure 2 displays the model-estimated probability of generating a bad metaphor as a function of levels of Gf and Openness to Experience. The negative slopes indicate that people are less likely to generate a bad metaphor as Gf and Openness increase, but the effects for Gf were stronger and more consistent. The effects of Gf were significant for the Gross Food (β = −0.28 [−0.50, −0.06], *p* = 0.011) and marginally significant for the Terrible Movie (β = −0.36 [−0.75, 0.03], *p* = 0.067) metaphors but not the Boring Class (−0.08 [−0.44, 0.27], *p* = 0.644). The effects of Openness to Experience, on the other hand, were consistently small and not significant (Terrible Movie: β = −0.13 [−0.46, 0.20], *p* = 0.434; Boring Class: β = −0.10 [−0.32, 0.11], *p* = 0.346; Gross Food: β = −0.13 [−0.29, 0.03], *p* = 0.108). Taken together, people low in Gf appear to be more likely to generate a bad metaphor, although the effects vary between the three prompts.

What were these bad metaphors like? As with the non-metaphors, we classified the bad metaphors into subtypes to gain insight into what makes a metaphor especially bad. Table 2 shows the prevalence of the different kinds, and Table 3 provides examples. The bad metaphors divided neatly into two types:

- *Exemplary Exemplar.* Many bad metaphors lacked semantic distance. The vehicle was an exemplar of the attributive category, but it was so exemplary that the metaphor was obvious, boring, and predictable. These responses tended to have the abstract structure “*x* is/is like the most salient and typical member of category *y*.”
- *Retrieved Cliché.* Other bad metaphors were stock phrases and dead metaphors retrieved from memory. These were often the most obvious possible idioms, such as describing being in a boring class as like “watching paint dry” or “watching grass grow,” given by participants who were presumably not trying to avoid clichés like the plague.

In short, the kinds of bad metaphors are familiar to creativity researchers who have worked with other open-ended tasks. Much like bad responses to unusual-uses tasks, the bad metaphor responses either lacked the semantic distance needed for novelty or were a pre-packaged response in memory sparked by the prompt.

## 4. Discussion

When you ask for open-ended creative metaphors, you get a startling variety of responses. Many participants will come up with clever, witty, and insightful metaphors; many more, however, will scrape the bottom of the cliché barrel. In the present research, we took a closer look at open-ended metaphor responses to understand the qualities of poor responses. The heterogeneity of responses is obscured by broad, continuous rating scales (e.g., *not at all creative* to *very creative*), and examining the varieties of wayward attempts to generate metaphors can illuminate important features of the cognitive processes involved when people try to create a clever metaphor.

The property attribution model (Glucksberg 2001) is a useful framework for understanding how creative metaphors go awry. The most salient distinction between poor responses was between *non-metaphors* (responses that did not meet the basic requirements of the metaphor generation prompt) and *bad metaphors* (responses that met the task requirements but lacked creativity). Although they were all failures to provide a basic, suitable response, non-metaphors were surprisingly varied. We discerned three kinds of non-metaphors. Some were failures to use figurative language—people described the topic adjectivally with the feature via adjectives instead of using categories and comparisons to transfer meaning from a vehicle to a topic. Others, however, failed to adhere to the basic task parameters—people described the wrong topic or attributed the wrong property to the right topic. 

Our intuition as creativity researchers interested in executive processes suggests some speculative explanations for the sources of these different kinds of failures. Adjective slips could have resulted from attempts to engage with the task, but the task’s difficulty—from understanding what metaphors are, identifying and using a higher-order category, and verifying that the response meets the task criteria—was too high. The “slip” could thus come from some combination of low linguistic knowledge and weak executive control, consistent with the roles of crystalized and fluid intelligence in creative metaphor (Beaty and Silvia 2013). The other two errors—using the wrong topic or the wrong property—we suspect result from hitting an impasse. Generating creative metaphors is challenging, and we suspect that many participants, when stuck and unable to come up with something suitable, just dash off any metaphor involving the topic (e.g., the wrong property responses) or any metaphor at all (e.g., unrelated clichés and song lyrics). These diverse kinds of failures are not apparent in other forms of creative ideation—like divergent thinking—because they have lower thresholds for response generation. In an unusual uses task, for example, people always have obvious, well-worn ideas at hand (e.g., use a brick to build a fireplace) that they can provide if stuck. Responses with the wrong topics and features are probably the “build a fireplace” of complex metaphor tasks. We should emphasize, however, that these are speculative ideas about the sources of these different kinds of poor responses, and they await future work that can test them directly. 

Bad metaphors, on the other hand, sorted into two kinds with strong parallels in other domains of creativity assessment. Some bad responses were semantically close: people selected the most salient exemplar of the attributive category—*garbage* for “gross things” or *torture* for “painful things”—as the vehicle for the metaphor. These metaphors were thus invariably apt and easy to understand, but they came across as trite and uninteresting. Most of them would be effective conventional metaphors. Other bad responses reflected memory retrieval. Memory is both a resource and a challenge for creative thought. Studies of people’s strategies for unusual uses tasks, for example, show that a common strategy is to search memory for uses one has observed and use them as responses (Gilhooly et al. 2007). Any idea that is directly recalled from memory tends to be highly accessible because it is familiar and obvious. For metaphor, these expressions tend to be highly conventionalized idioms that are, one might say, as dead as a doornail. For the first kind of bad metaphor, then, people created something unoriginal; for the second kind, they pulled a common idea from memory.

It is notable that fluid intelligence and Openness to Experience had different effects on the probability of generating non-metaphors and bad metaphors. For non-metaphors, both fluid intelligence and Openness predicted generating a real metaphor, so apparently one reason why these factors have predicted creative quality in past work is that people high in Gf and in Openness to Experience are simply more likely to come up with something suitable that adheres to the task requirements. For bad metaphors, however, Gf predicted generating a real metaphor for two of the three prompts—significant in one case, and marginal in the other—but Openness to Experience did not predict it for any of them. It is hard to unpack this finding further with the available data, but it is consistent with the view that creative cognition relies heavily on strategies that can be complex and executively demanding (Chrysikou 2019; Dygert and Jarosz 2020; Frith et al. in press; Lee and Therriault 2013; Nusbaum and Silvia 2011). Although correlated with Gf, Openness to Experience is more strongly connected to motivational aspects of creative thought, such as enjoying being creative, seeing oneself as a creative person, and pursuing creative goals (Karwowski and Lebuda 2016; Karwowski et al. 2019). Gf, in contrast, captures a cluster of skills associated with the capacity to coordinate and execute the processes needed to find, manipulate, and judge ideas. The finding that people high Gf were generally less likely to give a bad response is consistent with the view that Gf enables people to apply demanding strategies and manage the multiple processes involved in metaphor creation.

The present research is an initial look at wayward metaphors and an attempt to draw some speculative conclusions about how people tackle the complex task of generating creative metaphors. As a reanalysis of prior data, the present research is limited by the methods and measures used in prior work. The past studies were designed to assess variation in the creative quality of metaphors, so refined and expanded methods that can more explicitly focus on poor responses should be used in future research. As one example, the present methods are unable to discern real-but-bad metaphors with “wayward attributes” from responses intended to express sarcasm or irony (Skalicky 2020). This likely coarsens the analysis of such responses. Manipulating the kind of responses they should create (e.g., presence or absence of sarcasm) and asking people to explain their responses would be useful directions for future work. Metaphor creation seems like a problem that is perfect for protocol analysis and “think aloud” methods (Gilhooly et al. 2007). The poor responses are heterogeneous, representing a mix of failed attempts and bad attempts, which in turn are diverse and likely stem from interactions of knowledge, abilities, and task strategies. Collecting and analyzing verbal reports would yield valuable insight into how people manage the complex creative challenge that metaphors pose.

## Figures and Tables

**Table 1:** Intelligence measures included across metaphor samples.

Task	Description	Terrible Movie	Boring Class	Gross Food
Ravens Progressive Matrices	Select the item that completes a 3 × 3 matrix (12 min)		X	
Culture-Fair Matrix Completion	Select the item that completes a matrix (3 min)		X	
Culture-Fair Series Completion	Select the item that logically completes a series of shapes (3 min)	X	X	X
Letter Series	Discern the pattern governing sets of 4 letters, and pick the set that violates the rule (4 min)	X	X	X
Number Series	Discern the pattern underlying a series of numbers (4.5 min)	X	X	
Paper Folding	Select the item that represents what a square of paper would look like if folded and punched with holes (3 min)	X	X	X

Note: An X indicates that the intelligence task was included in the sample.

**Table 2:** Prevalence of non-metaphors and bad metaphors.

Metaphor Type		Terrible Movie	Boring Class	Gross Food
Non-Metaphors	Total of All Responses	11.7% (16 of 137)	15.4% (19 of 123)	6.0% (12 of 201)
	Adjective Slip	31.3% (5)	0% (0)	33.3% (4)
	Wayward Attribute	12.5% (2)	26.3% (5)	25.0% (3)
	Off-Topic Idiom	37.5% (6)	47.4% (9)	8.3% (1)
	Other	18.8% (3)	26.3% (5)	33.3% (4)
Bad Metaphors	Total of Metaphors	13.2% (16 of 121)	16.3% (17 of 104)	58.2% (110 of 189)
	Exemplary Exemplar	18.8% (3)	17.6% (3)	60.9% (67)
	Retrieved Cliché	50.0% (8)	58.8% (10)	0% (0)
	Other	31.3% (5)	23.5% (4)	39.1% (43)

Note: In each study, people generated one metaphor per prompt. The total number of responses generated varies for each prompt because of different sample sizes: terrible movie (*n* = 137), boring class (*n* = 123), and gross food (*n* = 201).

**Table 3:** Examples of non-metaphors and bad metaphors.

Response Type		Terrible Movie	Boring Class	Gross Food
**Non-Metaphors**	Adjective Slip	“That movie was sad” “Being in the theater was horrible”	*none*	“That drink was dirty” “Eating that was destructive” “It burned my throat and stomach”
	Wayward Attribute	“Sitting through that show was like going on an amazing and wonderful journey.” “Being in that theater was like a sunset on the beach”	“Sitting in that class was like laying out on a sunny day: HOTTT!!!” “That class was like a clown, humorous yet attention getting.”	“That coconut milk was like drinking strong sweet water”
	Off-Topic Idiom	“Relationships are like hot chocolate” “I miss you like an idiot misses the point”	“Paying my bill is like getting my period every month: Painful but all for a good cause.” “Love is like the sea in many aspects: it gets deeper and can send you in many directions.”	*Participant quoted lengthy song lyrics about self-harm*
**Bad Metaphors**	Exemplary Exemplar	“That movie was like torture” “That movies was like dying”	“Sitting in that class was like being in hell” “Being in that class was like hell, it was not fun at all.”	“That drink tasted like crap” “Drinking that was like tasting urine” “Eating that was like eating dirt” “Eating that is like actually tasting rotten eggs”
	Idiom Retrieval	“That movie hit me like a ton of bricks.” “Sitting through that show was like watching grass grow.”	“That class was like being forced to listen to nails on a chalkboard.” “Sitting through that class was like watching paint dry.”	*none*

## Appendix A. Task Instructions

For this task, you will be asked to come up with creative metaphors to describe things. As a background, there are a few common kinds of metaphors, and you can come up with any kind of them for this task.

A *simple metaphor* describes something by equating it to something else. Examples of simple metaphors are “All the world is a stage” (Shakespeare) and “A committee is a cul-de-sac down which ideas are lured and then quietly strangled” (Sir Barnett Cocks).

A *simile* (which is a kind of metaphor) describes something by saying it is like something else. An example of a simile is “Justice is like a train that is nearly always late” (Yevgeny Yevtushenko).

A *compound metaphor* has a second part that extends and explains the metaphor. Examples are “Life is like a box of chocolates: you never know what you’re going to get” (from the film “Forest Gump”) and “Men are like a fine wine: they all start out like grapes, and it’s our job to stomp on them and keep them in the dark until they mature into something you’d like to have dinner with” (Kathleen Mifsud).

You will be asked to come up with two metaphors. They can be any kind (a metaphor, a simile, or a compound metaphor). The aim is to come up with something creative—something clever, humorous, original, compelling, or interesting.

Think of the most boring high-school or college class that you have ever had: What was it like to sit through?

Please describe the experience with a metaphor. (If you need a start, you can try a beginning like “That class was…”, “Being in that class was like…”, or “Sitting through that class was like…”).

## References

[B1-jintelligence-09-00002] Ackerman Phillip L., Heggestad Eric D. (1997). Intelligence, personality, and interests: Evidence for overlapping traits. Psychological Bulletin.

[B2-jintelligence-09-00002] Beaty Roger E., Silvia Paul J. (2013). Metaphorically speaking: Cognitive abilities and the production of figurative language. Memory and Cognition.

[B3-jintelligence-09-00002] Beaty Roger E., Silvia Paul J., Benedek Mathias (2017). Brain networks underlying novel metaphor production. Brain and Cognition.

[B4-jintelligence-09-00002] Benedek Mathias, Jauk Emanuel, Fink Andreas, Koschutnig Karl, Reishofer Gernot, Ebner Franz, Neubauer Aljoscha C. (2014a). To create or to recall? Neural mechanisms underlying the generation of creative new ideas. NeuroImage.

[B5-jintelligence-09-00002] Benedek Mathias, Beaty Roger, Jauk Emanuel, Koschutnig Karl, Fink Andreas, Silvia Paul J., Dunst Beate, Neubauer Aljoscha C. (2014b). Creating metaphors: The neural basis of figurative language production. NeuroImage.

[B6-jintelligence-09-00002] Christensen Alexander P., Silvia Paul J., Nusbaum Emily C., Beaty Roger E. (2018). Clever people: Intelligence and humor production ability. Psychology of Aesthetics, Creativity, and the Arts.

[B7-jintelligence-09-00002] Christensen Alexander P., Cotter Katherine N., Silvia Paul J. (2019). Reopening openness to experience: A network analysis of four openness to experience inventories. Journal of Personality Assessment.

[B8-jintelligence-09-00002] Chrysikou Evangelia G. (2019). Creativity in and out of (cognitive) control. Current Opinion in Behavioral Sciences.

[B9-jintelligence-09-00002] Conner Tamlin S., Silvia Paul J. (2015). Creative days: A daily diary study of emotion, personality, and everyday creativity. Psychology of Aesthetics, Creativity, and the Arts.

[B10-jintelligence-09-00002] Costa Paul T., McCrae Robert R. (1992). Revised NEO Personality Inventory (NEO-PI-R) and NEO Five-Factor Inventory (NEO-FFI) Professional Manual.

[B11-jintelligence-09-00002] de Barros Débora P., Primi Ricardo, Miguel Fabiano Koich, Almeida Leandro S., Oliveira Ema P. (2010). Metaphor creation: A measure of creativity or intelligence?. European Journal of Education and Psychology.

[B12-jintelligence-09-00002] Dygert Sarah K., Jarosz Andrew F. (2020). Individual differences in creative cognition. Journal of Experimental Psychology: General.

[B13-jintelligence-09-00002] Frith Emily, Elbich Daniel, Christensen Alexander P., Rosenberg Monica D., Chen Qunlin, Silvia Paul J., Seil Paul, Beaty Roger E. Intelligence and creativity share a common cognitive and neural basis. Journal of Experimental Psychology: General.

[B14-jintelligence-09-00002] Gilhooly Kenneth J., Fioratou E. E., Anthony S. H., Wynn V. V. (2007). Divergent thinking: Strategies and executive involvement in generating novel uses for familiar objects. British Journal of Psychology.

[B15-jintelligence-09-00002] Glucksberg Sam (2001). Understanding Figurative Language: From Metaphors to Idioms.

[B16-jintelligence-09-00002] Glucksberg Sam (2003). The psycholinguistics of metaphor. Trends in Cognitive Sciences.

[B17-jintelligence-09-00002] Glucksberg Sam, Gibbs Raymond W. (2008). How metaphors create categories—Quickly. Cambridge Handbook of Metaphor and Thought.

[B18-jintelligence-09-00002] Goatly Andrew (2011). The Language of Metaphors.

[B19-jintelligence-09-00002] Karwowski Maciej, Lebuda Izabela (2016). The big five, the huge two, and creative self-beliefs: A meta-analysis. Psychology of Aesthetics, Creativity, and the Arts.

[B20-jintelligence-09-00002] Karwowski Maciej, Lebuda Izabela, Beghetto Ronald A., Kaufman James C., Sternberg Robert J. (2019). Creative self-beliefs. Cambridge Handbook of Creativity.

[B21-jintelligence-09-00002] Lee Christine S., Therriault David J. (2013). The cognitive underpinnings of creative thought: A latent variable analysis exploring the roles of intelligence and working memory in three creative thinking processes. Intelligence.

[B22-jintelligence-09-00002] Martin Demetri (2006). These Are Jokes [Audio Recording].

[B23-jintelligence-09-00002] Menashe Shay, Leshem Rotem, Heruti Vered, Kasirer Anat, Yair Tami, Mashal Nira (2020). Elucidating the role of selective attention, divergent thinking, language abilities, and executive functions in metaphor generation. Neuropsychologia.

[B24-jintelligence-09-00002] Nusbaum Emily C., Silvia Paul J. (2011). Are intelligence and creativity really so different? Fluid intelligence, executive processes, and strategy use in divergent thinking. Intelligence.

[B25-jintelligence-09-00002] Nusbaum Emily C., Silvia Paul J., Beaty Roger E. (2014). Ready, set, create: What instructing people to “be creative” reveals about the meaning and mechanisms of divergent thinking. Psychology of Aesthetics, Creativity, and the Arts.

[B26-jintelligence-09-00002] Nusbaum Emily C., Silvia Paul J., Beaty Roger E. (2017). Ha ha? Assessing individual differences in humor production ability. Psychology of Aesthetics, Arts, and Creativity.

[B27-jintelligence-09-00002] Pierce Russell S., Chiappe Dan L. (2009). The roles of aptness, conventionality, and working memory in the production of metaphors and similes. Metaphor and Symbol.

[B28-jintelligence-09-00002] Primi Ricardo (2014). Divergent productions of metaphors: Combining many-facet Rasch measurement and cognitive psychology in the assessment of creativity. Psychology of Aesthetics, Creativity, and the Arts.

[B29-jintelligence-09-00002] Silvia Paul J. (2006). Exploring the Psychology of Interest.

[B30-jintelligence-09-00002] Silvia Paul J., Beaty Roger E. (2012). Making creative metaphors: The importance of fluid intelligence for creative thought. Intelligence.

[B31-jintelligence-09-00002] Silvia Paul J., Nusbaum Emily C., Berg Christopher, Martin Christopher, O’Connor Alejandra (2009). Openness to experience, plasticity, and creativity: Exploring lower-order, high-order, and interactive effects. Journal of Research in Personality.

[B32-jintelligence-09-00002] Skalicky Stephen (2020). Exploring perceptions of novelty and mirth in elicited figurative language production. Metaphor and Symbol.

[B33-jintelligence-09-00002] Sutin Angelina R., Widiger Thomas A. (2017). Openness. Oxford Handbook of the Five Factor Model.

[B34-jintelligence-09-00002] Sutu Andrea, Phetmisy Cassandra N., Damian Rodica Ioana Open to laugh: The role of openness to experience in humor production ability. Psychology of Aesthetics, Creativity, and the Arts.

[B35-jintelligence-09-00002] Taylor Calvin W. (1947). A factorial study of fluency in writing. Psychometrika.

[B36-jintelligence-09-00002] Welhaf Matthew S., Smeekens Bridget A., Meier Matt E., Silvia Paul J., Kwapil Thomas R., Kane Michael J. (2020). Worst performance rule, or not-best performance rule? Latent-variable analyses of working memory capacity, mind-wandering propensity, and reaction time. Journal of Intelligence.

